# Factors associated with dropouts along the continuum of maternal care among adolescent girls in sub-Saharan Africa: A multilevel analysis of Demographic and Health Surveys from 15 countries

**DOI:** 10.4102/jphia.v17i1.1579

**Published:** 2026-07-31

**Authors:** Denise Kpebo, Tieba Millogo, Adama Baguiya, Eunice Chomi, Siaka Lougue, Seni Kouanda

**Affiliations:** 1 Department of Public Health, Medical School, University Allassane Ouattara, Abidjan, Côte d’Ivoire; 2 Department of Epidemiology and Biostatistics, Institut Africain de Santé Publique, Ouagadougou, Burkina Faso; 3 Maternal and Child Health Unit, National Public Health Institute, Abidjan, Côte d’Ivoire; 4 Department of Epidemiology and Biostatistics, Institute of Research in Health Sciences, Ouagadougou, Burkina Faso

**Keywords:** adolescence, continuum of maternal healthcare, maternal health, dropout, sub-Saharan Africa

## Abstract

**Background:**

Adolescent girls in sub-Saharan Africa (SSA) are at higher risk of maternal death as they tend to underuse maternal health services during pregnancy.

**Aim:**

This study aimed to identify the factors associated with dropouts along the continuum of maternal care (CoC) among adolescent girls.

**Setting:**

The analysis included 15 countries in SSA with high maternal mortality.

**Methods:**

We conducted a secondary data analysis using Demographic and Health Surveys data from 15 countries across SSA. All adolescent girls aged 15–19 years who reported a live birth in the 5 years preceding data each collection were included in the study. A multilevel logistic regression models were fitted to the data to identify the factors associated with dropouts.

**Results:**

Only 27.3% received all components of the CoC. Dropouts were highest between one and four antenatal care (ANC) visits (42.0%) and between childbirth and 48-h postnatal care (PNC) (37.4%). The odds of dropout between ANC1 and ANC4 visits were higher for adolescents living in rural areas, without formal education, from poor households and with poor ANC content (adjusted odds ratio: 1.68; 95% confidence interval: 1.51–1.86). Regarding 48-h PNC, the distance between home and health facility, the educational attainment and the content of ANC were associated with dropouts.

**Conclusion:**

Beside well-known socio-demographic factors, the study shows that poor quality of ANC services was a key driver of dropouts.

**Contribution:**

This study, which strengthens the body of evidence on maternal health among adolescent girls in SSA, could help in designing more efficient interventions.

## Introduction

Worldwide, around 11% of pregnancies occur in girls and young women aged 15–19 years. The vast majority of these (95%) occur in low- and middle-income countries.^1,2^ So far, the health of adolescents has been portrayed as being neglected. In the new era of the Sustainable Development Goals (SDGs), however, there have been increasing calls to reverse this trend.^3^ The increased interest in this vulnerable group is welcome and timely, given that complications related to pregnancy and childbirth are the leading cause of death among 15–19-year-old girls.^4,5^ Indeed, compared to older women, they have a higher risk of dying from complications related to pregnancy and childbirth.^4,6,7^ They are also at higher risk of severe morbidity, such as obstructed labour, haemorrhage, hypertension, low birth weight, preterm delivery, preeclampsia and anaemia.^4,5,7,8^

Sub-Saharan Africa (SSA) has the highest rate of maternal mortality in the world and the highest rate of early pregnancies.^2,7^ This implies that adolescent girls living in SSA are particularly exposed to the risk of maternal death. Research has shown that most of these deaths could be prevented by timely access and adequate use of maternal health services (MHS) because they offer an opportunity to prevent or manage the complications associated with pregnancy and childbirth. Antenatal care (ANC) is considered an essential MHS intervention to improve the survival and health of the mother-child dyad.^9,10,11^ Skilled attendance at childbirth (using a Skilled Birth Attendance or SBA), the delivery care provided by a skilled healthcare provider (midwife, doctor or nurse), is a key factor in reducing the risk of maternal death.^12,13^ Postnatal care for the mother (PNC), especially within 48 h of birth, is essential for the management of postnatal haemorrhage, one of the leading causes of maternal death in developing countries.^14,15^

Regarding the importance of these interventions, providing them as part of a continuum is likely to ensure maximum benefits for the mother-child dyad.^12^ The continuum of maternal care (CoC), from the prenatal to childbirth and post-partum periods, has become one of the key strategies to reduce maternal and newborn deaths and to improve the health and well-being of the mother-child dyad.^12^

Despite these proven benefits, adolescent girls tend to underuse MHS.^16,17,18^ Successful interventions to improve the use of the CoC by adolescent girls require a better understanding of both gaps in care-seeking along the way and the contributing factors.

Although a few studies have specifically examined the use of the CoC, most have focused on the general population of women of childbearing age.^19,20,21,22,23^ Regarding the specific sub-population of adolescent girls, most studies have treated the different components of maternal services (ANC, childbirth and PNC) as separate and independent entities.^17,18^

The objective of this study was to examine factors associated with dropout from the CoC among adolescent girls in SSA. This multicountry analysis focuses on four components of the CoC: (1) reception of at least one ANC visit, (2) reception of four ANC visits, (3) childbirth assisted by a skilled provider and (4) reception of PNC (for the mother) within 48 h of delivery. More specifically, this study was interested in:

- Assessing the utilisation of the CoC among adolescent girls.
- Measuring the proportion of dropouts at each stage of the CoC.
- Identifying the factors associated with dropouts at stages with the highest dropout rates.

## Research methods and design

### Source of data and study settings

This study used the most recent Demographic and Health Survey (DHS) data available, collected between 2014 and 2020, from 15 countries in SSA among those most affected by the scourge of maternal deaths (Online Appendix 1 Table 4-OA1).^24^ Data were extracted from the women’s survey datasets, obtained after authorisation from Measure DHS. Country selection was based on maternal mortality rates (MMR). Globally, 10 countries alone represent more than 59% of the maternal mortality worldwide. All SSA countries from this group, namely Nigeria, Sierra Leone, Ethiopia, Tanzania and Uganda, were included in our study. In addition, 18 of the 20 countries with the highest MMR worldwide are located in SSA.^24^ From these, we selected all countries with very high MMR (more than 500 maternal deaths per 100 000 live births) and included them in this analysis (Table 1).

**TABLE 1:** Demographic and health survey year per country.

Region	Country	Year of DHS survey
Western Africa	1. Guinea	2018
	2. Mali	2018
	3. Nigeria	2018
	4. Liberia	2019–2020
	5. Sierra Leone	2019
	6. Gambia	2019–2020
Central Africa	7. Chad	2014–2015
	8. Democratic Republic of the Congo	2014
	9. Cameroon	2018
Eastern and Southern Africa	10. Malawi	2015–2016
	11. Ethiopia	2016
	12. Uganda	2016
	13. Tanzania	2015–2016
	14. Kenya	2014
	15. Burundi	2016–2017

DHS, Demographic and Health Survey.

The DHS employ a cross-sectional design to collect data at household level from a nationally representative sample. These household surveys are conducted using a similar approach in all the countries: a multi-stage stratified cluster sampling design. Data on the use of MHS by women of childbearing age are reported for the last 5 years preceding the survey. The survey methodology, questionnaires, ethical considerations and reports are available online.^25^ A request to use the data was sent, and permission was obtained from the DHS Programme.

### Study population and sample size

The study population consisted of all participants who had at least one live birth in the 5 years preceding the survey and were aged 15–19 years at the time of the most recent birth. Adolescent girls who reported stillbirths were not included in this analysis because the DHS provides information about the use of MHS services only in the case of live births. For this secondary analysis, we virtually reconstituted the ‘cohort’ of adolescent girls, then, for the latest live birth, we followed the use of services along the CoC, from the first ANC to the receipt of the 48-h PNC, to assess the utilisation of the CoC and proportion of dropouts. Across all 15 countries, 142 706 women reported a live birth in the last 5 years, of whom 20 689 were aged 15–19 years at the time of birth. From this sub-population, 1848 had missing data that prevented us from categorising them with respect to our selected variables, so the analysis was restricted to 18 841 adolescent girls with complete data.

### Study variables

#### Outcome variable

The dependent variable was ‘dropout of the continuum of maternal care’, measured at four successive stages:

- Dropout before receipt of one ANC: At least one ANC, where dropout was coded ‘1’ for adolescent girls who did not receive any ANC visits and ‘0’ for those who received at least one ANC visit.
- Dropout before receipt of four ANC visits: Four ANC, where dropout was coded ‘1’ for adolescent girls who received at least one ANC, but who gave up before reaching the 4th ANC visit, and ‘0’ for those who received four ANC visits. Data for these variables were obtained from responses to three questions in the DHS: (1) Did you consult someone for prenatal care during this pregnancy? (2) Who did you consult? (3) How many ANCs did you receive during this pregnancy?
- Dropout before delivery with a SBA: Skilled attendance at childbirth (with a Skilled Birth Attendance or SBA), where dropout was coded ‘1’ for those who benefited from four ANC visits but did not continue with SBA, while those who continued with SBA after four ANC visits were coded ‘0’. For this variable, data came from responses to the DHS question: Who assisted you when ‘NAME’ was born? SBA was considered according to the DHS definition in each country, and this information is detailed in the Online Appendix 1 Table 1a-OA1.
- Drop out before receipt of PNC: Postnatal care within 48 h for the mother: those who benefited from four ANC and SBA but did not receive PNC within 48 h were considered dropouts and coded ‘1’; ‘0’ otherwise (received PNC within 48 h). The DHS contains questions about the type of provider and when the postnatal consultation was conducted.

For this variable, we included women who were examined by a health professional within 48 h of delivery, regardless of the location of the postpartum check-up. As done by previous authors,^22^ we categorised women’s responses regarding the timing of their first postpartum examination. We defined receipt of postpartum care within 48 h of birth as (1) whether the woman gave birth in a health facility, and declared to have been examined by a health professional in less than 48 h, before leaving the establishment; (2) gave birth in a healthcare facility, was not checked by a healthcare professional before discharge from the facility, but was checked after discharge by a healthcare professional within 48 h of birth; or (3) gave birth outside of a healthcare facility and reported being seen by a healthcare professional within 48 h of childbirth. In this analysis, we focused on PNC received within 48 h because the first 48 h of postpartum represent a critical period for the management of postpartum haemorrhage, which is one of the leading causes of maternal death in developing countries.^7,26^

Health professionals were considered skilled birth attendants per the country-specific definition, as described in the respective DHS reports (for the surveys under study) (Online Appendix 1 Table 2a-OA1 and Table 2b-OA1).

#### Independent variables

Based on the literature review^17,22,23,27^ and the availability of variables in the DHS dataset, we identified both individual and community-level independent variables to assess factors associated with dropout from the CoC. These were:

- Socio-demographic variables, notably, women’s level of education, household wealth index, exposure to mass media and employment status.
- Reproductive characteristics: marital status at the time of the survey, parity at the time of the most recent pregnancy and whether she wanted to become pregnant or not at the time of the most recent pregnancy.
  We have recoded some variables, based on the available categories in the DHS. The four categories of education variable were grouped into three groups, secondary level and tertiary level being grouped as ‘secondary and more’. For pregnancy desire, the categories ‘wanted but later’ and ‘did not want at all’ were combined into ‘not wanted’, leaving two categories for this variable (wanted vs. not wanted).
- Variables related to the health system: the timing of ANC and the ANC content score, which captures the content of care received during ANC visits (Online Appendix 1 Table 3-OA1 and Online Appendix 2 Table 1-OA2). For this score, we first considered the full range of services to be provided during ANC visits in each country, ranging from 7 to 10. Then, based on women’s self-reports of whether they received these services at least once at any point during their ANC visits, we calculated for each study participant the proportion of services received (based on the maximum available per country). From this, we calculated a tercile score and categorised it as follows: (1) lower tercile for a poor ANC score, (2) medium tercile for an average ANC score, and (3) upper tercile for a high ANC score.
- Community-level variables included place of residence and distance from the health facility. The country of residence was the variable used in the multilevel analysis.

The recoding process of the study variables, as well as the content of ANC services in each country, are all detailed in the Online Appendix 1 Table 2a-OA1, Table 2b-OA1, Table 3-OA1.

### Data analysis

Analyses were performed with Stata version 15, and all results were weighted and adjusted to account for survey multistage sampling design.

The descriptive stage was the first step in this analysis. The utilisation of any of the four services included in the continuum of care (at least one ANC, four ANC or more, SBA, PNC within 48 h) was presented for all countries and by region, using frequencies. As previously done by other authors,^19^ a visual illustration of the cascade of services included in the CoC made it possible to indicate the proportion of dropouts from one stage to another in the CoC.

Because DHS data were collected in nested units, we conducted a multilevel analysis using the xtmelogit command in Stata.

For the stages at which dropouts were highest, we first examined unadjusted associations between the CoC dropouts and each independent variable using simple logistic regression.

Covariates selection in the multivariable model was based on a statistical approach (*p* < 0.2 in univariate analysis). Then, building on the work of previous authors, we fitted four models for each stage.^28^ Firstly, an empty model, which is a baseline model with no explanatory variables, was fitted to assess the inherent variability in CoC dropout across clusters. Secondly, model 1 was fitted with community-level variables only and model 2 with individual-level variables only. Thirdly, model 3, including both individual-level and community-level variables, was fitted. Adjusted odds ratios (aOR) with 95% confidence interval (CI) were computed to measure the association between the independent variables and the CoC dropout. For each model, we used the intra-class correlation coefficients (ICC) to measure the proportion of total variance in CoC dropouts attributable to variation across clusters. The ratio of deviance was used to appreciate the model fitness, and the model with the lowest deviance was considered the best-fit model.

### Ethical considerations

This article followed all ethical standards for research without direct contact with human or animal subjects.

All DHS surveys received ethical clearance from both DHS Measure and the national ethics committees of each country. Thus, we did not look for additional ethical review for this secondary data analysis. However, before using these datasets, we requested and obtained formal authorisation from the DHS Programme.

## Results

### Socio-demographic and reproductive characteristics

A total of 18 841 respondents were included in this analysis, and their socio-demographic characteristics are summarised in Table 2. Most respondents (71.9%) lived in rural areas, and about a quarter (24.1%) had no education. Nearly half of the respondents (45.6%) were from poor households, and 57.1% had no exposure to any mass media. In addition, distance to a health facility was an issue for nearly half of the adolescent girls (46.9%). About three out of five respondents (59.9%) received their first ANC in the second trimester of pregnancy, and less than a third (27.5%) had a high score of ANC content.

**TABLE 2:** Socio-demographic and reproductive characteristics of the study population.

Variables	*n*	Weighted %
**Country**		
Gambia	636	2.8
Guinea	934	5.0
Liberia	843	4.0
Nigeria	1748	9.5
Sierra Leone	1148	6.0
Mali	1056	5.6
Cameroon	987	5.0
Chad	1174	6.9
Democratic Republic of the Congo	1728	8.8
Burundi	640	3.6
Uganda	1619	8.6
Tanzania	1050	6.2
Ethiopia	876	4.4
Kenya	1879	9.8
Malawi	2523	13.8
Total	18 841	100.0
**Level of education**		
No education	4757	24.1
Primary	8392	45.1
Secondary +	5692	30.8
Total	18 841	100.0
**Household wealth index**		
Poorest	5025	26.7
Poorer	4032	21.4
Middle	3860	20.5
Richer	3620	19.2
Richest	2304	12.2
Total	18 841	100.0
**Marital status**		
Married	13 718	72.6
Not married	5123	27.4
Total	18 841	100.0
**Exposure to media**		
Yes	7909	42.9
No	10 932	57.1
Total	18 841	100.0
**Parity**		
One child	13 443	72.2
Two children +	5398	27.8
Total	18 841	100.0
**Currently working**		
Yes	9065	53.2
No	9776	46.8
Total	18 841	100.0
**Pregnancy desire**		
Wanted	12 789	67.5
Not wanted	6048	32.5
Total	18 841	100.0
**Timing of first ANC visit†**		
1st trimester	5770	32.3
2nd trimester	10 208	59.9
3rd trimester	1367	7.8
Total	17 345	100.0
**ANC content score†**		
Poor ANC score	5283	31.0
Average ANC score	7260	41.5
High ANC score	4802	27.5
Total	17 345	100.0
**Type of place of residence**		
Urban	5202	28.1
Rural	13 639	71.9
Total	18 841	100.0
**Distance to health facility**		
Big problem	8923	46.9
Not a big problem	9918	53.1
Total	18 841	100.0

ANC, antenatal care.

† , Only among those who achieved at least one ANC visit.

### Utilisation and dropout from the continuum of maternal care

**FIGURE 1:**
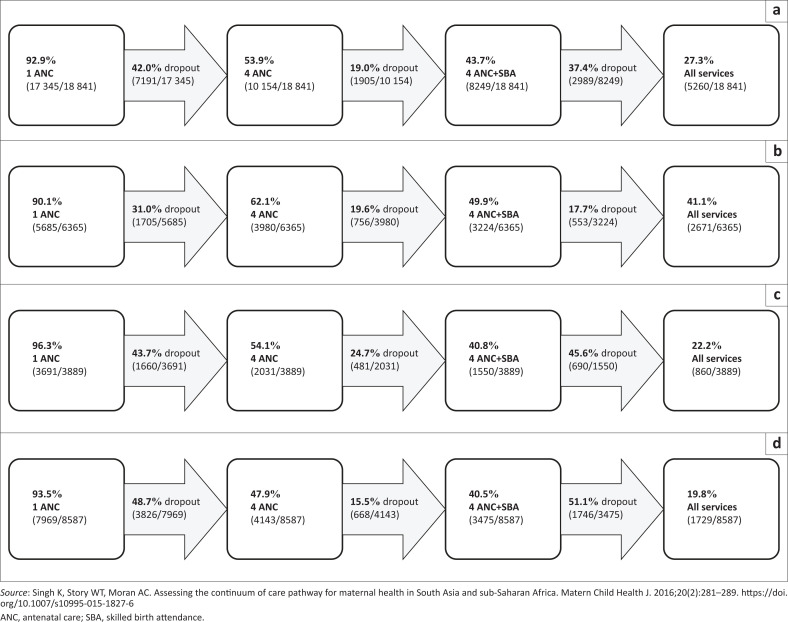
Utilisation and dropout of the continuum of maternal healthcare (*weighted percentage*). (a) In all 15 countries (*N* = 18 841), (b) In Western Africa (*n* = 6365), (c) In Central Africa (*n* = 3889), (d) In Eastern and Southern Africa (*n* = 8587).

Figure 1 presents the proportion of adolescent girls who remained in the continuum of care from one stage to another, as well as the evolution of dropouts across these stages. Out of 18 841 adolescent girls, 92.9% achieved one ANC visit, 53.9% achieved four or more ANC visits and 48.7% achieved both 4 ANC visits and a childbirth with an SBA. Finally, fewer than 3 out of 10 adolescent girls (27.3%) used all services in the CoC (Figure 1a).

Regarding dropout trends, among 17 345 adolescent girls who received at least one ANC visit, 42.0% dropped out before completing four or more ANC visits. Among those who received four ANC visits (*n* = 10 154), 19.0% dropped out and did not have an SBA at childbirth. Among those who continued with SBA at childbirth (*n* = 8249), 37.4% dropped out of the CoC without receiving any PNC within 48 h after delivery (Figure 1a).

At the regional level, dropouts were highest both for ANC4 (48.7% in Eastern Africa and 43.7% in Central Africa) and 48-h PNC (51.1% in Eastern Africa and 45.6% in Central Africa) (Figure 1d).

Generally, the countries that performed poorly, with at least 50% of dropouts at both stages, were from Eastern and Southern Africa and from Central Africa. The countries that performed relatively well were in Western Africa (see Online Appendix 2 Table 1-OA2).

### Factors associated with dropouts of the continuum of maternal care

#### Factors associated with dropouts between the first and the fourth antenatal care visits

At the community level, the odds of dropout were higher among adolescent girls living in rural areas (aOR: 1.21; 95% CI: 1.10–1.29) compared to those living in urban areas (Table 3). With regard to individual-level factors, adolescent girls with no education (aOR: 1.17; 95% CI: 1.04–1.32) and those with primary level education (aOR: 1.20; 95% CI: 1.09–1.31), from poorest (aOR: 1.30; 95% CI: 1.10–1.40), and poor households (aOR: 1.16; 95% CI: 1.07–1.35), and those for whom the index pregnancy was not desired (aOR: 1.21; 95% CI: 1.11–1.31), were more likely to drop out of the CoC. In addition, the odds of dropout were also higher among adolescent girls who had a poor ANC score (aOR: 1.68; 95% CI: 1.51–1.86) and an average ANC score (OR: 1.18; 95% CI: 1.07–1.30), compared to those with a high ANC score.

**TABLE 3:** Individual and community-level factors associated with dropouts from the continuum of maternal care between antenatal care 1 and antenatal care 4, multivariable analysis.

Variable	Null model		Model 1		Model 2		Model 3	
	aOR	95% CI	aOR	95% CI	aOR	95% CI	aOR	95% CI
**Residence**								
Rural	-	-	1.42	1.19–1.70	-	-	1.21	1.10–1.29†
Urban	-	-	1	-	-	-	1	-
**Distance to get to health facility**								
Big problem	-	-	1.13	1.05–1.21	-	-	1.04	0.97–1.32
Not a big problem	-	-	1	-	-	-	-	-
**Level of education**								
No education	-	-	-	-	1.26	1.12–1.41	1.17	1.04–1.32†
Primary	-	-	-	-	1.21	1.10–1.33	1.20	1.09–1.31†
Secondary +	-	-	-	-	1	-	1	-
**Household wealth index**								
Poorest	-	-	-	-	1.30	1.19–1.42	1.22	1.10–1.40†
Poorer	-	-	-	-	1.12	1.01–1.23	1.16	1.07–1.35†
Medium	-	-	-	-	1.27	1.15–1.41	1.19	1.06–1.33†
Richer	-	-	-	-	1.31	1.11–1.51	1.05	0.87–1.22
Richest	-	-	-	-	1	-	1	-
**Exposure to mass media**								
Yes	-	-	-	-	1	-	1	-
No	-	-	-	-	1.15	1.06–1.25	1.13	1.04–1.23†
**Working status**								
Not working	-	-	-	-	1.08	1.00–1.16	1.08	0.99–1.16†
Working	-	-	-	-	1	-	1	-
**Parity**								
1	-	-	-	-	1	-	1	-
2+	-	-	-	-	1.11	1.03–1.21	1.13	1.04–1.23
**Pregnancy desire**								
Wanted	-	-	-	-	1	-	1	-
Not wanted	-	-	-	-	1.22	1.13–1.32	1.21	1.11–1.31
**Timing of ANC**								
1st trimester	-	-	-	-	1	-	1	-
2nd trimester	-	-	-	-	3.52	3.24–3.83	3.52	3.23–3.84†
3rd trimester	-	-	33.05	27.2–40.20	32.9	27.1–40.22	-	-
**ANC content score**								
Poor ANC score	-	-	-	-	1.70	1.54–1.87	1.68	1.51–1.86†
Average ANC score	-	-	-	-	1.19	1.09–1.31	1.18	1.07–1.30
High ANC score	-	-	-	-	1	-	-	-
**Random parameters and model comparison**								
Community-level variance	0.48	-	0.25	-	0.47	-	0.15	-
ICC	12.76	6.59–23.29	10.24	8.02–16.37	12.61	6.51–23.05	9.6	7.25–16.11
MOR	1.94	1.84–1.99	1.86	1.36–2.26	1.93	1.85–2.02	1.76	1.24–1.99

Note: Random parameters and model comparison: PCV % – Null model = 1; Model 1 = 0.50; Model 2 = 4.2; Model 3 = 68.8. –2LLR – Null model = 22276.786; Model 1 = 22109.898; Model 2 = 18270.5282; Model 3 = 18251.3208.

ANC, antenatal care; ICC, intra-class correlation coefficients; aOR, adjusted odds ratio; MOR, median odds ratio; PCV, proportional change in variance; LLR, likelihood ratio.

† , Indicate that the results were significant in the multilevel models.

The ICC in the null model indicated that the difference across countries accounted for 12.76% of the total variation in dropouts from four or more ANCs.

#### Factors associated with dropouts between childbirth with skilled birth attendance and the receipt of 48-h postnatal care

For adolescent girls who used the CoC until childbirth with SBA, only the variables related to the level of education, the working status and the content of ANC visits were associated with the outcome of interest (Table 4). For instance, compared to adolescent girls with a high ANC content score, the odds of dropout were more than two-fold higher among those with a poor ANC score (aOR: 2.50; 95% CI: 2.15–2.91), and 57% higher among those with an average score.

**TABLE 4:** Individual and community-level factors associated with dropouts from the continuum of maternal care between skilled birth attendance and 48-h postnatal care, multivariable analysis.

Variable	Null model		Model 1		Model 2		Model 3	
	AOR	95% CI	AOR	95% CI	AOR	95% CI	AOR	95% CI
**Residence**								
Rural	-	-	1.24	1.07–1.43	-	-	1.15	0.98–1.22
Urban	-	-	1	-	-	-	1	-
**Distance to get to health facility**								
Big problem	-	-	1.12	1.07–1.25	-	-	1.09	0.98–1.22
Not a big problem	-	-	1	-	-	-	1	-
**Level of education**								
No education	-	-	-	-	1.21	1.04–1.42	1.20	1.02–1.42†
Primary	-	-	-	-	1.13	1.00–1.28	1.13	0.99–1.28
Secondary +	-	-	-	-	1	-	1	-
**Working status**								
Not working	-	-	-	-	1	-	1	-
Working	-	-	-	-	1.14	1.02–1.26	1.17	1.05–1.30†
**Pregnancy desire**								
Wanted	-	-	-	-	1	-	1	-
Not wanted	-	-	-	-	1.11	0.99–1.23	1.12	1.00–1.25
**Timing of ANC**								
1st trimester	-	-	-	-	1	-	1	-
2nd trimester	-	-	-	-	0.98	0.88–1.09	0.97	0.87–1.08
3rd trimester	-	-	0.97	0.60–1.55	0.99	0.62–1.60	-	-
**ANC content score**								
Poor ANC score	-	-	-	-	2.59	2.23–3.00	2.50*	2.15–2.91†
Average ANC score	-	-	-	-	1.59	1.40–1.80	1.57	1.39–1.78†
High ANC score	-	-	-	-	1	-	1	-
**Random parameters and model comparison**								
ICC	17.76	6.59–23.29	14.85	6.9–21.2	14.28	8.75–24.23	11.79	6.90–26.07
MOR	2.23	1.84–3.99	2.06	1.24–1.99	2.02	1.85–2.02	1.80	1.36–3.26

Note: Random parameters and model comparison: Community-level variance – Null model = 0.61; Model = 10.25; Model 2 = 20.54; Model 3 = 30.22. PCV % – Null model = 1; Model 1 = 10.59; Model 2 = 20.115; Model 3 = 30.639. –2LLR – Null model = 9638.3866; Model 1 = 8902.179; Model 2 = 8960.6528; Model 3 = 8721.9494.

ANC, antenatal care; ICC, intra-class correlation coefficients; AOR, adjusted odds ratio; MOR, median odds ratio; PCV, proportional change in variance.

† , Indicate that the results were significant in the multilevel models.

In this multilevel analysis, again, the ICC in the null model implied that the difference across countries accounted for 17.76% of the variation in dropouts between childbirth with SBA and 48-h PNC.

## Discussion

This study was conducted to quantitatively assess the CoC among adolescent girls, measure the extent of dropout and identify factors associated with dropout. The results show that only 3 out of 10 adolescent girls (27.3%) received all maternal services in SSA. Dropouts were more frequent between ANC1 and ANC4 visits (42.0%) and between childbirth with SBA and the 48-h PNC visit (37.4%). Socio-demographic and economic factors, as well as the content of ANC visits, were associated with these dropouts.

Such a low use of the CoC by adolescent girls is in agreement with the findings of many other studies in SSA,^21,22,23,27^ and remains a worrying situation, considering the high rate of maternal mortality in this group.^7^ Each element of the maternal healthcare continuum provides essential and life-saving benefits.^12,29,30,31^ Poor CoC coverage could explain why interventions with proven effectiveness do not appear to have the expected impact on adolescent maternal mortality in SSA.

The results of this study show that a high proportion of dropouts (42.0%) occur before adolescents reach four or more ANC visits. These results are similar to those of a multicountry study in SSA, which showed that up to 40% of adolescent girls also dropped out before this stage.^27^ Frequent contact with healthcare providers is likely to increase clients’ health awareness and access to key health information, and could thus partially explain the link between four or more ANC visits and the use of the CoC.^28,30^ Sub-Saharan African countries should promote strategies and interventions to increase adolescent girls’ use of these services.

A high proportion of dropouts (37.4%) was also observed between childbirth and receipt of 48-h PNC. Regardless of maternal age, low PNC use appears to be common.^26^ Postnatal care service is one of the crucial interventions to reduce both maternal and neonatal mortality, as the postnatal period is the most life-threatening period for mothers and newborns. Yet, PNC service utilisation remained generally low in developing countries, as women viewed it as needed only when there was a problem with the mother or newborn.^32,33^ In addition, studies on adolescent girls’ use of MHS highlighted providers’ negligence towards adolescents, partially explained by societal stigma often associated with adolescent girls’ pregnancy.^16,34^ It is likely that our study population could have experienced such issues. Interventions aimed at reducing the toll of maternal deaths in this sub-population should be focused on improving providers’ perception and attitude towards adolescents. This study showed significant disparities in the use of the CoC, thereby reaffirming the effects of socio-economic variables such as level of education, place of residence, and household wealth index.^22,28^ Programmes to increase girls’ education are a long-term solution for increased use of the continuum. In the short term, for maternal and child health to progress more rapidly, it may be necessary to design programmes that allow uneducated girls from poor households to have unrestricted access to maternal healthcare. In addition, a qualitative approach could provide a deep understanding of the socio-economic challenges and obstacles specifically encountered by this vulnerable sub-population, as well as identify levers for the successful implementation of effective interventions.

Among adolescent girls who received antenatal care and skilled birth assistance, few factors were associated with the subsequent discontinuation of PNC. Other studies in SSA and other low-income countries have also shown that few factors explain dropouts between childbirth and receipt of 48-h PNC.^19,20,35,36^ However, the importance of factors related to health services, such as the content of ANC services, is noted. It has been observed that dropouts from 48-h PNC are greater among adolescent girls who received fewer services during ANC visits. Evidence shows that the frequency and quality of prenatal care influence the retention of women in the CoC.^16,20^ Yet, adolescent girls are not only likely to have fewer visits but also to benefit from lower-quality ANC compared to older women.^16^

The main strength of this study lies in the national representativeness of the data analysed for each country. The results can be generalised, offering an opportunity to inform programmes and interventions at the regional level.

However, there are some limitations that we cannot rule out. Firstly, there may be recall bias because women were asked to recall events that occurred in the 5 years preceding the survey. Secondly, women who did not survive their pregnancy or childbirth, and pregnancies that resulted in stillbirth, were not included. Thirdly, the DHS does not collect data on pregnancy complications, while adolescent girls with pregnancy complications may receive more comprehensive ANC care, resulting in higher ANC content.

Despite these limitations, the results of this study provide insights into ways to strengthen the utilisation of the CoC, suggesting some significant policy implications.

Firstly, there is a need to implement policies and programmes that will increase adolescents’ completion of the CoC, rather than merely the use of its specific components. Policies and programmes implemented to increase women’s awareness of the need to complete the CoC must consciously make room for adolescent girls’ particular needs and challenges. These could be community and provider involvement sessions to raise awareness of how to support adolescents’ quest to complete the CoC. Secondly, the association between ANC content and the use of the CoC emphasises the need for greater commitment to improve the quality of maternal care for adolescent girls. Indeed, the new WHO recommendations indicate that pregnant women should receive at least eight ANC contacts to improve maternal and newborn health outcomes.^37^ In efforts to adopt these new guidelines, countries in SSA must pay particular attention to the content and quality of maternal care for adolescent girls, rather than the mere increase in the number of ANC visits. Thirdly, the association between socio-economic factors such as education and wealth index with the use of the CoC suggests that efforts should be made to enhance long-term interventions to increase adolescents’ educational attainment. In the short term, for maternal and child health to progress more rapidly, it may be necessary to design programmes that allow uneducated girls from poor households to have unrestricted access to the CoC.

## Conclusion

The use of the CoC was low among adolescent girls, with the highest dropouts between one and four antenatal visits and between the delivery and reception of PNC. Factors associated with dropouts from the CoC included level of education, place of residence, household wealth index and content of antenatal care visits. Programmes promoting maternal health should focus on accessing and using the full continuum rather than merely the utilisation of specific components. The content of antenatal care is associated with the use of the continuum and should be further strengthened in adolescent girls who come into contact with the health system.
